# The overexpression of peroxiredoxin-4 affects the progression of idiopathic pulmonary fibrosis

**DOI:** 10.1186/s12890-019-1032-2

**Published:** 2019-12-30

**Authors:** Tetsuya Hanaka, Takashi Kido, Shingo Noguchi, Sohsuke Yamada, Hirotsugu Noguchi, Xin Guo, Aya Nawata, Ke-Yong Wang, Keishi Oda, Tsutomu Takaki, Hiroto Izumi, Hiroshi Ishimoto, Kazuhiro Yatera, Hiroshi Mukae

**Affiliations:** 1 Department of Respiratory Medicine, School of Medicine, University of Occupational and Environment Health, Japan, 1-1 Iseigaoka, Yahatanishiku, Kitakyushu City, Fukuoka, 807-8555 Japan; 2 Department of Pathology and Laboratory Medicine, Kanazawa Medical University, 1-1 Daigaku, Uchinada, Kahoku, Ishikawa 920-0293 Japan; 3 Department of Pathology, Field of Oncology, Graduate School of Medical and Dental Sciences, Kagoshima University, 8-35-1 Sakuragaoka, Kagoshima, 890-8544 Japan; 4 Department of Pathology and Cell Biology, School of Medicine, University of Occupational and Environmental Health, Japan, 1-1 Iseigaoka, Yahatanishiku, Kitakyushu City, Fukuoka, 807-8555 Japan; 5 Shared-Use Research Center, University of Occupational and Environmental Health, Japan, 1-1 Iseigaoka, Yahatanishiku, Kitakyushu City, Fukuoka, 807-8555 Japan; 6 Department of Occupational Pneumology, Institute of Industrial Ecological Sciences, University of Occupational and Environmental Health, Japan, 1-1 Iseigaoka, Yahatanishiku, Kitakyushu City, Fukuoka, 807-8555 Japan; 7 Department of Respiratory Medicine, Unit of Translational Medicine, Nagasaki University Graduate School of Biomedical Sciences, 1-12-4 Sakamoto, Nagasaki, 852-8523 Japan

**Keywords:** Acute exacerbation, Idiopathic pulmonary fibrosis, Mice, Peroxiredoxin 4

## Abstract

**Background:**

Acute exacerbation of idiopathic pulmonary fibrosis (AE-IPF) is life-threatening. Several serum biomarkers, such as Krebs von den Lungen-6 (KL-6) and surfactant protein D (SP-D), are clinically used for evaluating AE-IPF, but these biomarkers are not adequate for establishing an early and accurate diagnosis of AE-IPF. Recently, the protective roles of the members of the peroxiredoxin (PRDX) family have been reported in IPF; however, the role of PRDX4 in AE-IPF is unclear.

**Methods:**

Serum levels of PRDX4 protein, KL-6, SP-D and lactate dehydrogenase (LDH) in 51 patients with stable IPF (S-IPF), 38 patients with AE-IPF and 15 healthy volunteers were retrospectively assessed using enzyme-linked immunosorbent assay. Moreover, as an animal model of pulmonary fibrosis, wild-type (WT) and PRDX4-transgenic (Tg) mice were intratracheally administered with bleomycin (BLM, 2 mg/kg), and fibrotic and inflammatory changes in lungs were evaluated 3 weeks after the intratracheal administration.

**Results:**

Serum levels of PRDX4 protein, KL-6, SP-D and LDH in patients with S-IPF and AE-IPF were significantly higher than those in healthy volunteers, and those in AE-IPF patients were the highest among the three groups. Using receiver operating characteristic curves, area under the curve values of serum PRDX4 protein, KL-6, SP-D, and LDH for detecting AE-IPF were 0.873, 0.698, 0.675, and 0.906, respectively. BLM-treated Tg mice demonstrated aggravated histopathological findings and poor prognosis compared with BLM-treated WT mice. Moreover, PRDX4 expression was observed in alveolar macrophages and lung epithelial cells of BLM-treated Tg mice.

**Conclusions:**

PRDX4 is associated with the aggravation of inflammatory changes and fibrosis in the pathogenesis of IPF, and serum PRDX4 may be useful in clinical practice of IPF patients.

## Background

Idiopathic pulmonary fibrosis (IPF) is a fibrosing parenchymal lung disease that has chronic, progressive, and even fatal outcomes. The natural history of patients with IPF is extremely complex; moreover, the clinical course of such patients varies from relatively stable to sudden acute exacerbation, which often prove fatal. Recently, clinical trials have demonstrated that antifibrotic agents, such as pirfenidone and nintedanib, reduce the decline in forced vital capacity (FVC) and prolong progression-free survival in patients with IPF [1, 2]; however, the prognosis of IPF is still poor with an estimated median survival time of 2–5 years after the initial diagnosis [3].

Approximately 5–10% of patients with IPF generally demonstrate acute exacerbation (AE) annually [4, 5], leading to very high mortality [6]. An early diagnosis of AE-IPF is the most important clinical parameter; however, available data regarding useful biomarkers to precisely predict progressive patients with IPF are limited. Therefore, simple and effective diagnostic biomarkers for detecting AE-IPF are required for prompt decision making in the proper treatment of patients with IPF. Several serum biomarkers, such as Krebs von den Lungen-6 (KL-6) and surfactant proteins A and D (SP-A and -D, respectively), are clinically used for diagnosing AE-IPF [7, 8], but these markers are not adequately efficient; other biomarkers, such as monomeric periostin [9] and heat shock protein 47 (HSP47) [10], have also been recently reported as surrogate markers for detecting AE-IPF.

Peroxiredoxin (PRDX) is a recently identified antioxidant family that contains reactive cysteine in a conserved region near the N-terminus [11, 12]. Six members of the PRDX family have been identified in mammals (PRDX1–6). Human PRDX4 is the only secretory isoform that exists in both intra- and extracellular spaces [13, 14] and is ubiquitously synthesized and is abundantly expressed in various organisms [15]. Regarding the role of each PRDX in IPF, a protective role of PRDX1 in bleomycin (BLM)-induced pulmonary fibrosis (PF) mice was reported [16]. In addition, the co-localization of PRDX2 with platelet-derived growth factor receptors (PDGFRs) and proliferating cells in human lung tissue in patients with IPF/usual interstitial pneumonia (UIP) was also reported [17]. In addition, an increase in the PRDX4 mRNA expression in the lung tissue of patients with interstitial lung disease was reported [18], but the role of PRDX4 in the pathogenesis and progression of IPF is still unclear.

We previously generated human PRDX4-transgenic mice (Tg mice) using C57BL/6 mice and reported that PRDX4 may have a protective role against the progression of atherosclerosis and nonalcoholic fatty liver disease via its antioxidant effect [19, 20].

In the present study, we compared the serum PRDX4 protein level in patients with stable IPF (S-IPF), AE-IPF and healthy volunteers to evaluate the significance of PRDX4 in patients with IPF. In addition, we examined the pathogenetic roles of PRDX4 in pulmonary inflammation and fibrosis using Tg mice in a BLM-induced PF model.

## Methods

### Human study

S-IPF and AE-IPF were diagnosed based on the criteria for IPF and AE-IPF [4, 21, 22], respectively. The serum samples obtained from patients with S-IPF and AE-IPF between April 2010 and December 2016 were analyzed for serum PRDX4 protein level. In addition, serum samples of 15 healthy adult volunteers (32–47 years old) with no medical histories were also collected. This study was conducted according to the Declaration of Helsinki and was approved by the Ethics Committee of Medical Research, University of Occupational and Environmental Health, Japan (approval number H29–182). For patients with S-IPF in whom AE-IPF occurred during the follow-up period, the serum samples were obtained both in the stable state of IPF (as S-IPF) prior to AE-IPF and at the time of AE of IPF (as AE-IPF). Clinical data such as age, sex, body mass index (BMI), and smoking history; clinical manifestations; and laboratory data including serum KL-6, SP-D, and lactate dehydrogenase (LDH) levels were collected.

### Serum and bronchoalveolar lavage fluid (BALF) PRDX4 protein levels in humans and mice

Serum and BALF PRDX4 protein levels in both humans and mice were assessed using enzyme-linked immunosorbent assay (ELISA) (Abnova, Taipei, Taiwan) according to the manufacturer’s protocol as previously described [20].

### Animal study

The animal study was approved by the Ethics Committee of Animal Care and Experimentation, University of Occupational and Environmental Health, Japan (approval number AE-14-019) and performed in accordance with the National Institutes of Health guidelines. Male wild-type (WT) mice (C57BL/6, 10-week-old) and Tg mice (weight, 21–28 g) were selected and maintained on a regular diet (CE-2, CLEA Japan, Inc., Tokyo, Japan). WT mice were obtained from Kyudo Co., Ltd. (Tosu, Japan). PRDX4-Tg mice were generated and provided in our facility [23].

### Intratracheal BLM treatment in mice

2.0-mg/kg BLM (Nippon Kayaku, Tokyo, Japan) was dissolved in 40-μL sterile saline. This solution (BLM group) or 40-μL sterile saline alone (saline group) was intratracheally instilled in both WT and Tg mice after receiving intraperitoneal sodium pentobarbital. Body weights were recorded at 0, 3, 7, 14 and 21 days after the intratracheal instillation. On day 21, the mice were inhaled with 3% sevoflurane to initiate anesthesia and then deeply anesthetized with intraperitoneal injection of sodium pentobarbital (50 mg/kg). These mice were euthanized by cutting the inferior vena cava to induce exsanguination after collecting blood from the inferior vena cava. Separately, a group sacrificed on day 0 without any intratracheal instillation was provided in order to evaluate the human PRDX4 mRNA and protein levels at baseline (baseline group).

The numbers of mice were as follows: WT mice at baseline (WT-baseline) (*n* = 5), Tg mice at baseline (Tg-baseline) (*n* = 5), saline-treated WT mice (WT-saline) (*n* = 5), saline-treated Tg mice (Tg-saline) (*n* = 5), BLM-treated WT mice (WT-BLM) (*n* = 14) and BLM-treated Tg mice (Tg-BLM) (*n* = 14). The numbers of mice that survived until day 21 among the saline- and BLM-treated mice were as follows: WT-saline (*n* = 5), Tg-saline (*n* = 5), WT-BLM (*n* = 12) and Tg-BLM (*n* = 7). Furthermore, to avoid the influence of BAL on other experimental results, the following numbers of mice were prepared for the BALF analysis: WT-baseline (*n* = 5), Tg-baseline (*n* = 5), WT-saline (n = 5), Tg-saline (*n* = 5), WT-BLM (*n* = 14) and Tg-BLM (*n* = 14). The number of mice that survived until day 21 among the saline- and BLM-treated mice for the BALF analysis were as follows: WT-saline (*n* = 5), Tg-saline (*n* = 5), WT-BLM (*n* = 12) and Tg-BLM (*n* = 6).

### Microscopic computed tomography in mice

Under general anesthesia induced by inhaling sevoflurane, microscopic computed tomography (micro-CT) images of mouse lungs were evaluated on day 21 after instillation using a micro-CT system (CosmoScan GX, Rigaku Co., Tokyo, Japan) using the following conditions: 90 kV, 88 μA; field of view, 36 mm; voxel size, 60 × 60 × 60 μm; and scan time, 4 min.

### BALF in mice

BALF in mice was obtained by cannulating the trachea using a 20-gage catheter and by washing three times using 1-mL sterile saline. Cytospin was performed to evaluate the presence of BALF cells, and the obtained cell-free supernatants were stored at − 80 °C until PRDX4 protein assessment as previously described [20].

### Histopathological and immunostaining assessments of murine lungs

Left lungs of the mice were removed by incising at the anterior midline, were fixed with 15% formalin neutral buffer solution (Wako, Osaka, Japan) at 25 cmH_2_O, and were embedded in paraffin. Subsequently, 3-μm sections of embedded lung tissues were stained with hematoxylin and eosin (HE) and Masson’s trichrome. The Ashcroft score was assessed to evaluate PF as previously described [24]. Each specimen was independently scored by two observers (TH and WKY), including a histopathologist, and the mean scores were considered as the fibrotic score.

The mouse anti-human monoclonal fibronectin antibody (1:100; Abcam, Cambridge, United Kingdom), rabbit anti-human PRDX4 polyclonal antibody (1:500; BioReagents, Golden, CO, USA) [19, 23, 25], and anti-mouse monoclonal antibody for 8-hydroxy-2′-deoxyguanosine (8-OHdG) (1:100; Japan Institute for the Control of Aging, Fukuroi, Japan) were used. The number of positively stained cells in five randomly selected fields per section was quantified (original magnification: × 200) as previously described [19, 23] in analyses of 8-OHdG.

### Double immunofluorescence staining of murine lungs

For immunofluorescence studies, the sections of right lung of Tg mice were embedded in the OCT compound (Sakura Finetek Japan, Tokyo, Japan), snap-frozen in liquid nitrogen, and stored at − 80 °C until use. To identify PRDX4-positive cells in lung tissues, 6-μm-thick cryosections were used for double immunofluorescence staining of polyclonal rabbit anti-human PRDX4 antibody (1:500; Thermo Fisher Scientific, Yokohama, Japan) and were visualized using goat anti-rabbit IgG antibodies conjugated with Alexa Fluor 488® (green; Thermo Fisher Scientific, Yokohama, Japan) combined with monoclonal rat anti-mouse Mac-2 (1:500; Cedarlane Laboratories, Burlington, Canada), monoclonal mouse anti-rat thyroid transcription factor (TTF-1; 1:100; Dako Cytomation Co., Tokyo, Japan), and monoclonal mouse anti-human α-smooth muscle actin (α-SMA; 1:150; Dako Cytomation Co., Tokyo, Japan) antibodies visualized using goat anti-mouse IgG antibodies conjugated with Alexa Fluor 546® (red; Thermo Fisher Scientific, Yokohama, Japan).

### Real-time polymerase chain reaction (PCR)

The total RNA extracted from the homogenized right lung tissue using ISOGEN reagent (Nippon Gene, Tokyo, Japan) was reverse-transcribed. The expression of CC chemokine ligand 2 (CCL-2), collagen 1A1, connective tissue growth factor (CTGF), human PRDX4, interferon γ (IFN-γ), interleukin (IL)-1β, IL-4, IL-6, IL-13, IL-17A, tumor necrosis factor (TNF)-α, platelet-derived growth factor subunit B (PDGF-B), active tissue growth factor-β1 (TGF-β1), fibronectin and glyceraldehyde-3-phosphate dehydrogenase (GAPDH) was quantified using real-time quantitative PCR with the ABI prism 7000 sequence detection system (Applied Biosystems, Foster City, CA, USA), as previously described [20, 26]. The relative expression level of each gene was normalized to that of GAPDH using random primers as previously reported [27].

### Fibronectin protein concentration in murine lungs

Fibronectin protein concentrations in mouse lung homogenates were measured using the ELISA kit (Abbexa, Cambridge, United Kingdom) according to the manufacturer’s protocol.

### Statistical analysis

Data are presented as medians (interquartile range) or the number of subjects (%) in human data and as means (standard error of the mean) in murine data, unless otherwise specified. Continuous variables were compared using the Mann–Whitney U test or Kruskal-Wallis test, and categorical variables were compared using the chi-square test or Fisher’s exact test, as appropriate. Changes in human serum PRDX4 protein, KL-6, SP-D, and LDH levels were determined using the Wilcoxon signed rank test. The receiver operating characteristic (ROC) curves and Youden indices were used to determine the optimal cut-off of serum PRDX4, KL-6, SP-D, and LDH levels to distinguish AE-IPF from S-IPF. The survival probability of each group was estimated using the Kaplan–Meier method and compared using the global log-rank test. Values of *P* <  0.05 were considered to be statistically significant. All calculations were performed using the StatFlex software version 6 (Artech, Osaka, Japan).

## Results

### Clinical characteristics of patients with IPF

We analyzed the blood samples of 51 patients with S-IPF, 38 patients with AE-IPF and 15 healthy volunteers. Among them, BALF samples were also evaluated in 14 S-IPF and 10 AE-IPF patients. During the observation period, 9 of the 51 S-IPF patients developed AE, and the serum samples of these 9 patients were collected in both the stable state before AE developed (as S-IPF) and also at time of AE development (as AE-IPF). Table 1 shows the characteristics of the enrolled patients. Compared with the S-IPF group, the AE-IPF group showed a significantly lower arterial partial pressure of oxygen/fraction of inspired oxygen (PaO_2_/FiO_2_) ratio, significantly higher alveolar–arterial difference of oxygen (A-aDO_2_), significantly lower FVC and significantly lower diffusing capacity of the lungs for carbon monoxide (DL_CO_), and the overall survival of the AE-IPF group was significantly worse than that of the S-IPF group after the diagnosis.

**Table 1:** Characteristics of healthy volunteers and patients with S-IPF and AE-IPF

	HV (*n* = 15)	S-IPF (*n* = 51)	AE-IPF (*n* = 38)	Three groups, *p-value*	HV/S-IPF, *p-value*	HV/AE-IPF, *p-value*	S-IPF/AE-IPF, *p-value*
Age, years	36 (34–40)	72 (68–75)	72 (64–75)	< 0.001	< 0.001	< 0.001	0.983
Sex, male	13 (87)	39 (76)	35 (92)	0.217	0.396	0.542	0.051
BMI	22.9 (20.9–24.8)	22.9 (20.7–24.7)	22.9 (21.1–25.8)	0.785	0.786	0.875	0.480
Smoking history, never/former/current	15/0/0	12/30/9	2/31/5	< 0.001	< 0.001	< 0.001	0.254
PaO_2_/FiO_2_ ratio, mmHg	–	395 (324–429)	220 (128–296)	–	–	–	< 0.001
A-a DO_2_, mmHg	–	14 (9–31)	135 (58–251)	–	–	–	< 0.001
FVC^a^, L	–	2.00 (1.43–2.66)	1.85 (1.73–2.31)	–	–	–	0.674
FVC^a^, % predicted	–	67 (55–79)	54 (47–68)	–	–	–	0.039
DL_CO_^b^, % predicted	–	66 (43–83)	37 (29–49)	–	–	–	0.005
Overall survival^c^, days	–	1059	57	–	–	–	< 0.001

Data are presented as median (interquartile range) or n (%)

^a^The numbers of patients with FVC measured were as follows: S-IPF (*n* = 51), AE-IPF (*n* = 15)

^b^The numbers of patients with DL_CO_ measured were as follows: S-IPF (*n* = 35), AE-IPF (*n* = 10)

^c^The date of baseline of overall survival is the day of diagnosis of S-IPF and AE-IPF

Abbreviations: *AE-IPF* Acute exacerbation of idiopathic pulmonary fibrosis, *A-a DO*_*2*_ Alveolar-arterial difference of oxygen, *BMI* Body mass index, *DL*_*CO*_ Diffusing capacity of the lungs for carbon monoxide, *FVC* Forced vital capacity, *HV* Healthy volunteer, *PaO*_*2*_/FiO_*2*_ Arterial partial pressure of oxygen/fraction of inspired oxygen, *S-IPF* Stable idiopathic pulmonary fibrosis

### Comparison of serum PRDX4 protein, KL-6, SP-D, and LDH levels

**Fig. 1:**
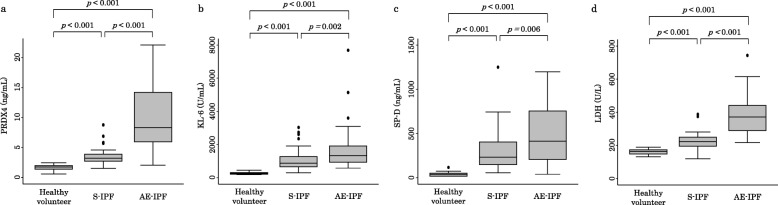
Serum PRDX4 protein, KL-6, SP-D and LDH levels in healthy volunteers and patients with S-IPF and AE-IPF. **a**, **b**, **c**, and **d**) Serum PRDX4 protein, KL-6, SP-D and LDH levels were significantly higher in S-IPF patients than those in healthy volunteers. In addition, these levels in AE-IPF patients were significantly higher than those in patients with S-IPF

The serum levels of PRDX4 protein, KL-6, SP-D and LDH in patients with S-IPF were all significantly higher than those in healthy volunteers (Fig. 1), and those in AE-IPF patients were all significantly higher than those in S-IPF patients. In contrast, the BALF PRDX4 protein level did not differ significantly between the patients with S-IPF and AE-IPF (Additional file 1: Figure S1), nor were any significant correlations noted between the serum and BALF PRDX4 protein levels in either case (Additional file 2: Figure S2).

### Changes in serum PRDX4 protein, KL-6, SP-D, and LDH levels in patients with S-IPF that subsequently progressed to AE-IPF

**Fig. 2:**
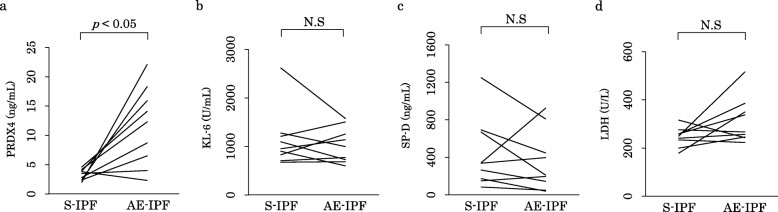
Changes in serum PRDX4, KL-6, SP-D, and LDH levels in patients with S-IPF and AE-IPF. **a**) A significant increase in serum PRDX4 protein level was observed at AE-IPF diagnosis compared with that at S-IPF diagnosis. **b**, **c**, and **d**) The increase in serum KL-6, SP-D, and LDH level at AE-IPF diagnosis was not significant compared with that at S-IPF diagnosis

Nine patients with S-IPF subsequently progressed to AE-IPF, and the interval until the diagnosis from S-IPF to AE-IPF ranged from 62 to 1373 (median: 552) days. For these patients, changes in serum PRDX4 protein, KL-6, SP-D, and LDH levels at S-IPF and AE-IPF were compared. Serum PRDX4 protein levels at AE-IPF were significantly higher than those at S-IPF (*p* <  0.05) (Fig. 2a); however, serum KL-6, SP-D, and LDH levels showed no significant changes (Figs. 2b, c, and d).

### The significance of serum PRDX4 protein, KL-6, SP-D, and LDH levels in AE-IPF using ROC curves

**Fig. 3:**
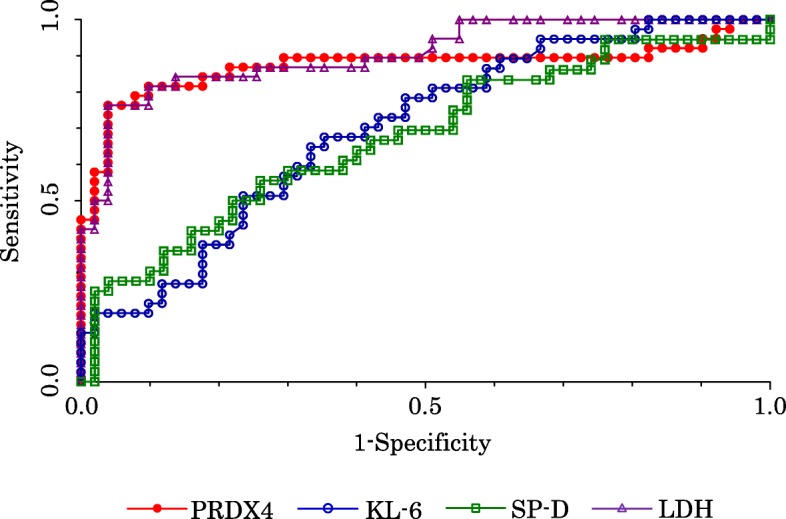
ROC curve of serum PRDX4, KL-6, SP-D, and LDH for diagnosing AE-IPF. The AUCs using ROC curve were 0.873, 0.698, 0.675, and 0.906 of serum PRDX4, KL-6, SP-D, and LDH, respectively, for diagnosing AE-IPF

The area under the curves (AUCs) of serum PRDX4 protein, KL-6, SP-D, and LDH in AE-IPF were 0.873, 0.698, 0.675, and 0.906, respectively (Fig. 3). The optimal cut-off levels (Youden index) of serum PRDX4 protein, KL-6, SP-D, and LDH were 5.84 ng/mL, 1046 U/mL, 374 ng/mL, and 281 U/L, respectively. The sensitivities and specificities of serum PRDX4 protein, KL-6, SP-D, and LDH levels were 0.763 0.676, 0.556, and 0.763 and 0.961, 0.647, 0.740, and 0.961, respectively.

### Survival rates and body weight changes after BLM treatment in mice

**Fig. 4:**
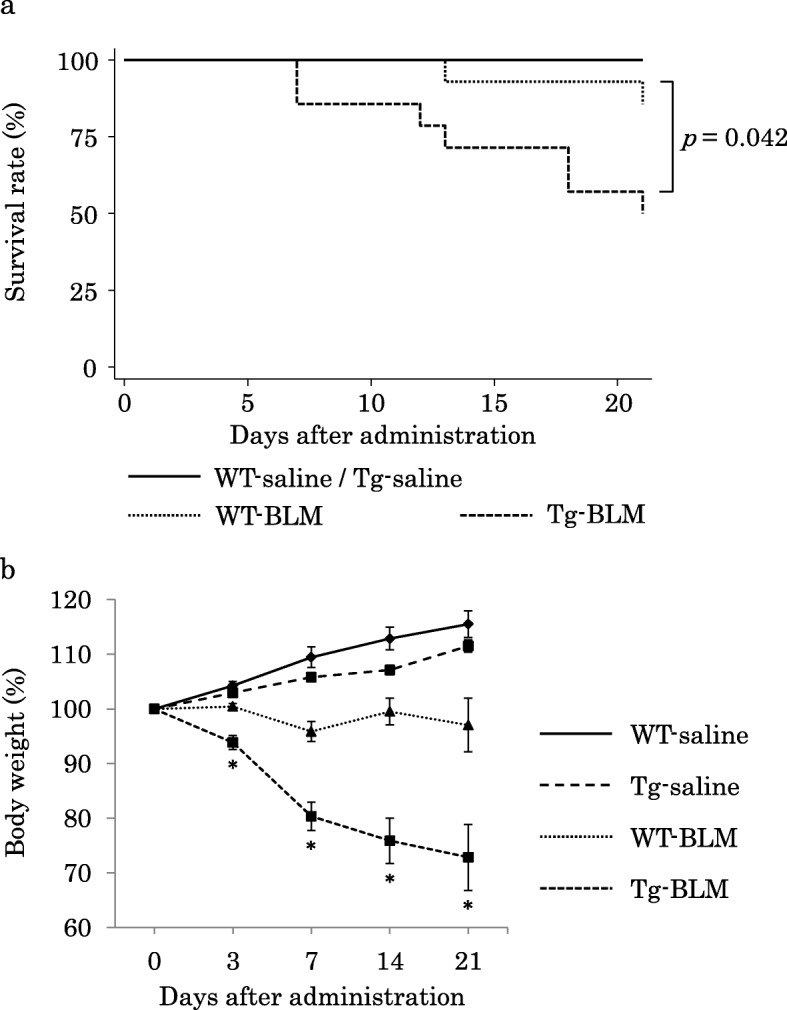
Survival rates and temporal changes in body weight until 21 days after BLM or saline administration in mice. **a**) The survival rates of Tg-BLM were worse than those of WT-BLM. **b**) Tg-BLM showed a significant loss in body weight compared with WT-BLM. * *P* <  0.05

Figure 4a shows the survival time of mice until day 21 after the intratracheal administration of BLM or saline. The survival time of Tg-BLM was significantly lower than that of WT-BLM (*p* = 0.042). In addition, the body weights of Tg-BLM significantly decreased compared with those of WT-BLM (Fig. 4b).

### Human PRDX4 expression in the murine lung

**Fig. 5:**
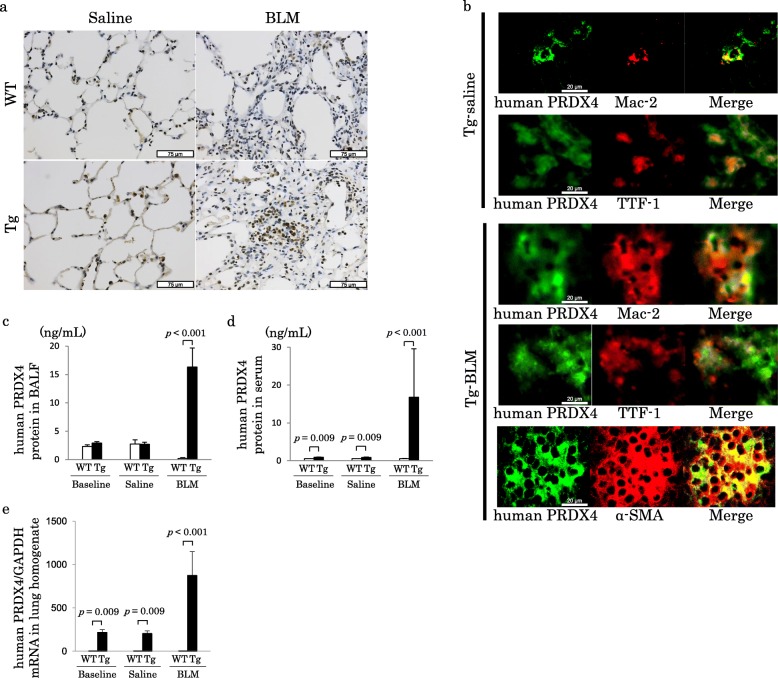
PRDX4 expression in mice. **a**) Immunohistochemical staining of murine lung for detecting PRDX4. PRDX4-positive cells, indicating PRDX4 expression, were stained brown. **b**) Double immunohistochemical staining of murine lung. Human PRDX4 (cytoplasm is green stained) and Mac-2 (cytoplasm is red stained), human PRDX4 (cytoplasm is green stained), and TTF-1 (nucleus is red stained) are expressed in same cells in Tg-saline. Human PRDX4 (cytoplasm is green stained) and Mac-2 (cytoplasm is red stained), human PRDX4 (cytoplasm is green stained) and TTF-1 (nucleus is red stained), human PRDX4 (cytoplasm is green stained) and α-SMA (cytoplasm is red stained) are expressed in the same cells in Tg-BLM. **c** and **d**) An ELISA for assessing the BALF and serum human PRDX4 protein levels. The BALF and serum human PRDX4 protein levels were markedly higher in Tg-BLM than in WT-BLM. The serum human PRDX4 protein levels in Tg-baseline and Tg-saline mice were significantly higher than in the WT mice. **e**) The human PRDX4 mRNA levels in murine lung homogenates. The human PRDX4 mRNA levels were markedly higher in Tg-baseline, Tg-saline and Tg-BLM than in WT mice

The immunohistochemistry of PRDX4 demonstrated the presence of PRDX4-positive cells in WT and Tg mice. The intensity and distribution of PRDX4 in Tg-BLM were greater than those in WT-BLM (Fig. 5a).

Double immunofluorescence staining of the lung sections of Tg mice (Fig. 5b) demonstrated that human PRDX4 and Mac-2, human PRDX4 and TTF-1 were expressed in the same cells in Tg-saline and Tg-BLM. Moreover, human PRDX4 and α-SMA were expressed in the same cells in Tg-BLM. These results suggest that human PRDX4 is mainly localized in alveolar macrophages and epithelial cells in the lungs of Tg mice.

In addition, the BALF human PRDX4 protein level in Tg-BLM was significantly higher than in WT-BLM (Fig. 5c). The serum human PRDX4 protein level in Tg-baseline, Tg-saline and Tg-BLM was significantly higher than in the WT mice (Fig. 5d). Furthermore, the human PRDX4 mRNA levels in lungs of Tg-baseline, Tg-saline and Tg-BLM were significantly higher than in the WT mice (Fig. 5e).

### Quantification of PF in murine lungs

**Fig. 6:**
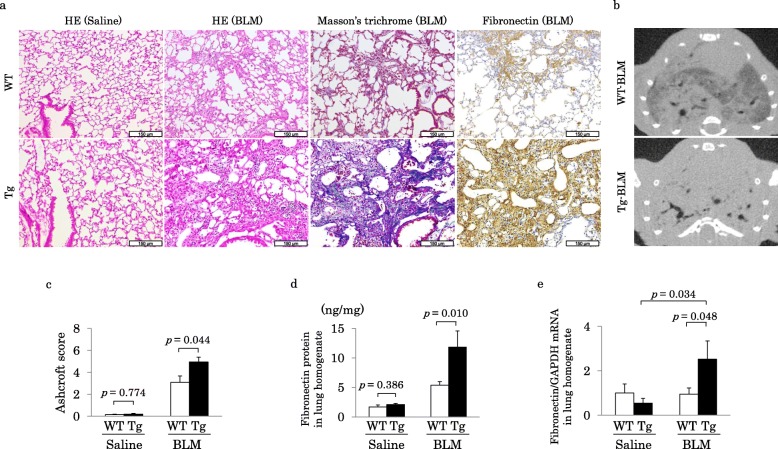
Analyses of lung inflammation and fibrosis in mice. **a**) Representative pathological photographs of lung tissue of WT or Tg mice 21 days after the administration of saline or BLM. Lung inflammation and fibrosis in Tg-BLM were greater than those in WT-BLM. **b**) Consolidation and traction bronchiectasis were observed in both WT-BLM and Tg-BLM at day 21 using micro-CT. These findings were more extensively observed in Tg-BLM than in WT-BLM. **c**) Ashcroft score 21 days after the administration of saline or BLM. The Ashcroft score in Tg-BLM was significantly higher than that in WT-BLM. **d**) The protein level of fibronectin at 21 days was significantly higher in Tg-BLM than that in WT-BLM. **e**) The mRNA level of fibronectin at 21 days was significantly higher in Tg-BLM than that in WT-BLM

Representative pathological findings from murine lung tissues following HE, Masson’s trichrome, and immunohistochemical staining with anti-fibronectin antibody 21 days after BLM or saline treatment are shown in Fig. 6a. HE staining of lung tissues of both WT-saline and Tg-saline was normal whereas that of lung tissues of WT-BLM and Tg-BLM demonstrated pulmonary fibrotic changes in both WT and Tg mice. Masson’s trichrome and immunohistochemical staining revealed more intense collagen deposition and strong immunostaining of fibronectin in Tg-BLM than in WT-BLM (Fig. 6a). Chest micro-CT demonstrated traction bronchiectasis and severe consolidations in Tg-BLM compared with those in WT-BLM (Fig. 6b).

Ashcroft scores in both WT-saline (0.2 ± 0.0) and Tg-saline (0.2 ± 0.1) were almost normal; however, these scores in Tg-BLM were significantly higher than those in WT-BLM (4.9 ± 0.4 vs. 3.1 ± 0.6, respectively; *p* = 0.044) (Fig. 6c).

Both protein and mRNA levels of fibronectin in lung homogenates of Tg-BLM were significantly higher than those in lung homogenates of WT-BLM (Figs. 6d and e).

### Quantitative analysis of proinflammatory cytokines and growth or profibrotic factor expressions in murine lungs

**Fig. 7:**
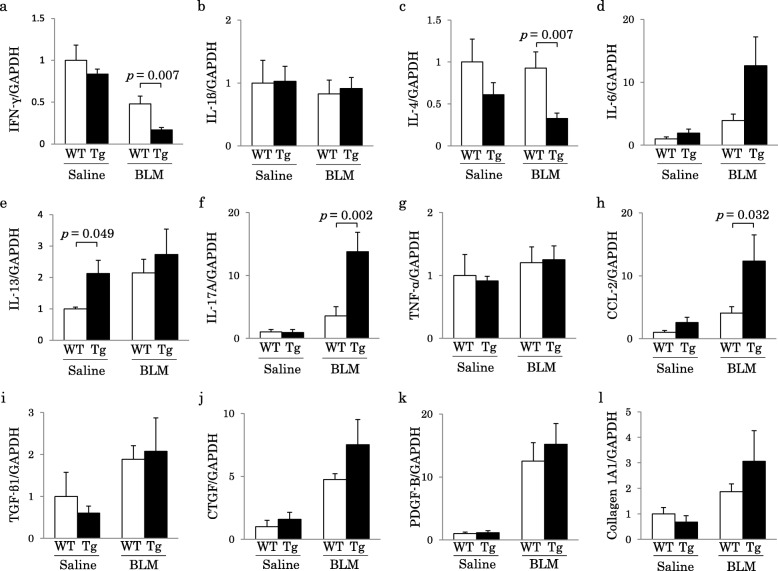
mRNA expression in murine lung homogenates. **a**, **b**, **c**, **d**, **e**, **f**, **g**, **h**, **i**, **j**, **k**, and **l**) The mRNA expression of molecules related to inflammation and fibrosis in lung homogenates was evaluated using real-time PCR. The mRNA levels of IL-17A in Tg-BLM were significantly higher than those in WT-BLM

The IL-13 mRNA expression in the lung was significantly higher in Tg-saline than in WT-saline mice, but no marked differences were noted in the IL-1β, IL-6, TNF-α, CCL-2, IL-17A, TGF- β1, CTGF, PDGF-B or collagen 1A1 expression. In Tg-BLM mice, the pulmonary mRNA levels of CCL-2 and IL-17 were significantly higher while those of IL-4 and IFN-γ were significantly lower than in WT-BLM mice. However, no significant differences were found in other assessed cytokines or in growth or profibrotic factors between these two groups (Fig. 7).

### Oxidative stress analysis

**Fig. 8:**
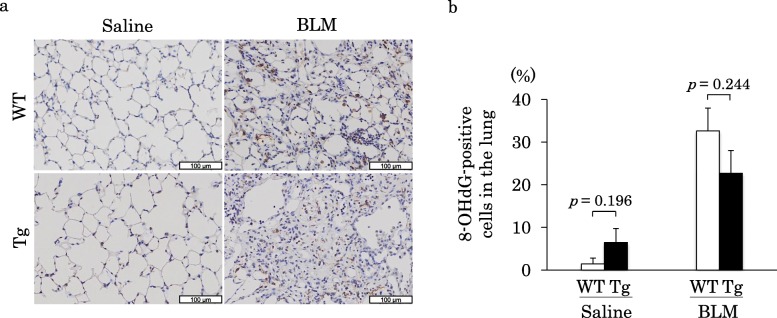
Analyses of 8-OHdG in murine lungs. **a**) Immunohistochemical staining for 8-OHdG in WT and Tg mice lungs 21 days after the administration of saline or BLM. Immunohistochemical staining for 8-OHdG in the cytoplasm to brown indicates positivity. **b**) The percentage of 8-OHdG-positive cells 21 days after the administration of saline or BLM. Increase in the proportion of 8-OHdG positive cells was not significant between Tg-BLM and WT-BLM

Immunohistochemical staining of murine lung tissues (Fig. 8a) demonstrated that the proportion of 8-OHdG-positive cells significantly increased in the BLM-treated group compared with that in the saline-treated group of both WT and Tg mice. However, the proportion of 8-OHdG-positive cells in WT-BLM and Tg-BLM was not significantly different (Fig. 8b).

## Discussion

In the present study, we investigated the role and significance of PRDX4 in PF. In human study, the patients with AE-IPF had higher serum PRDX4 protein levels than those with S-IPF, and serum PRDX4 protein levels showed higher prognostic value than both serum KL-6 and SP-D levels for distinguishing AE-IPF from S-IPF. In addition, animal studies using a BLM-induced PF model of Tg mice demonstrated that Tg-BLM had significantly worse pulmonary fibrotic changes with significantly poor survival rates compared with WT-BLM mice.

Generally, patients with IPF are well known to exhibit elevated serum KL-6, SP-D, and LDH levels, and these levels are also recognized as predictive biomarkers of survival of these patients [4]. However, a Japanese report showed that the AUCs for serum KL-6, SP-D, and LDH levels for distinguishing AE-IPF from S-IPF were 0.576, 0.718, and 0.84, respectively [28]. Moreover, other reports showed that serum KL-6 and SP-D levels did not differ between patients with S-IPF and AE-IPF [10, 29], suggesting poor presence of potential biomarkers for distinguishing AE-IPF from S-IPF [10]. Similar to previous reports [10, 29], serum KL-6, SP-D, and LDH levels at AE-IPF were not significantly higher than those at S-IPF in this study. Conversely, serum PRDX4 protein and LDH levels showed better AUC profiles, obtained using ROC curves, to distinguish AE-IPF from S-IPF than serum KL-6 and SP-D levels. A recent report demonstrated that HSP47 (47 kDa) may be a potential biomarker for distinguishing AE-IPF from S-IPF, and monomeric periostin (90 kDa) and latent TGF-β binding protein-2 (195–240 kDa) are useful for predicting poor prognosis in patients with IPF [9, 30]. The molecular weight of these biomarkers is smaller than that of KL-6 (> 200 kDa) [31], and considering that the molecular weight is associated with biomarker profiles [10, 29], the small molecular weight of PRDX4 (34 kDa) [20] may explain its better profile as a marker for detecting AE-IPF. However, the molecular size of SP-D is 43 kDa, which is not considerably different from that of PRDX4. Recently, monomeric periostin was reported to be expressed in fibroblastic foci, and KL-6 and SP-D were expressed in regenerating alveolar type II cells, and these different release sites may partly explain the profiles of these biomarkers [9]. Further investigations are therefore warranted to elucidate the release sites of PRDX4 in patients with IPF.

PRDX4 is widely expressed in various organs other than the lungs. Moreover, elevated serum PRDX4 protein levels are associated with poor outcomes and high mortalities in patients with sepsis [32, 33]. The role of PRDX4 in pulmonary inflammation and fibrosis is still unclear, and the specificity of serum PRDX4 in patients with AE-IPF as a biomarker could not be evaluated in this study. However, increased serum PRDX4 level was associated with an aggravation of pulmonary inflammatory changes, fibrosis, and poor prognosis in the murine model; therefore, elevated serum PRDX4 levels observed in patients with AE-IPF may originate from increased PRDX4 expression in the lungs. Further studies are necessary to elucidate the clinical significance of serum PRDX4 levels in AE-IPF and other respiratory disorders.

In the present study, the mRNA levels of proinflammatory cytokines such as IL-1β, IL-6 and TNF-α in murine lung homogenates showed no significant differences between WT- and Tg-BLM. These proinflammatory cytokines are generally used as markers of the acute phase of PF in BLM-induced PF murine model [34], and these results may be influenced by the timing of evaluation. In contrast, significantly increased gene expressions of IL-17A and fibronectin were observed in the lung tissues of Tg-BLM compared with those in the lung tissues of WT-BLM. Reportedly, IL-17A is involved in the pathogenesis of BLM-induced PF [35], and early IL-17A axis leads to pulmonary inflammation and fibrosis in the late phase [36]. In addition, danger signals (damage-associated molecular patterns), which induce an immune response by acting on the dendritic cells, also cause tissue injury and inflammation that are mediated by IL-17A [37, 38]. Therefore, the PRDX4-induced overexpression of IL-17A may play an important role in the pathogenesis and progression of BLM-induced pulmonary inflammation and fibrosis. The expression of NF-κB-regulated cytokines in the WT and Tg mice after saline or BLM challenge was not markedly different in the present study, but both the suppressive effect of PRDX4 on NF-κB [13] and the activating effect of IL-17A on NF-κB [39] might partly explain the conflicting findings seen in the expression of NF-κB-regulated cytokines in the WT and Tg mice after saline or BLM treatment.

The oxidant–antioxidant imbalance plays an important role in the pathogenesis of IPF [40], and an increased expression of 8-OHdG, a marker of oxidative stress, is observed in the lungs of patients with IPF [41]. The expressions of 8-OHdG in the lungs of Tg-BLM and WT-BLM were not different in the present study. Kikuchi et al. reported that mice lacking PRDX1, a member of the PRDX family, exhibited aggravated lung inflammation and fibrosis due to an increase in the pulmonary oxidant stress [16]; moreover, Wang et al. demonstrated that a lack of PRDX6 resulted in lung injury in mice [42]. In addition, we previously reported regarding the antioxidant effects of PRDX4 in Tg mice in the models of diabetes mellitus [23], atherosclerosis [16], and nonalcoholic fatty liver disease [25]. Conversely, extracellular PRDXs, such as PRDX1, PRDX2, PRDX5 and PRDX6 induce severe inflammation in the brain by functioning as danger signals in brain injury models [38]; thus, conflicting actions of PRDXs have been reported in several inflammatory diseases. Although the mechanisms of the protective roles of PRDX4 are still unknown, stimulated danger signals including inflammatory cytokines, such as IL-6 and IL-8, other than profibrotic cytokines may play an important role in the pathogenesis and progression of AE in patients with IPF [43]. Our results suggest that the overexpression of PRDX4 in the lung may exert an exacerbating effect on pulmonary fibrosis by inducing inflammatory cytokines as danger signals rather than a protective effect as an antioxidant enzyme in the acute to subacute phase of pulmonary inflammation; however, further investigation regarding this is necessary.

Among the members of the PRDX family, PRDX1 is expressed in alveolar macrophages in the BLM-induced PF murine model [16]. However, the PRDX4 expression in the normal and inflamed human lungs is still unclear, and the types of cells that secrete PRDX4 as well as the ratio of secretion and intracellular PRDX4 in each cell type have been unclear in patients with IPF. In the present study, immunohistochemistry of murine lungs demonstrated the PRDX4 expression in alveolar macrophages and alveolar epithelial cells in Tg-BLM, although the amount and ratio of secreted and intracellular PRDX4 in each cell type remained unclear. This location is similar to that of the PRDX1 expressed in fibrotic murine lungs [16].

This study has several limitations. First, the human study was a single-center retrospective study with a limited number of patients with S-IPF and AE-IPF for detecting serum and BALF PRDX4 protein levels. Second, the backgrounds such as age, gender and smoking histories of the healthy volunteers and those of IPF patients were not matched. Third, we were unable to assess changes between baseline and follow-up period in pulmonary function, because many patients did not undergo a pulmonary function test during the follow-up period, therefore, we could not evaluate the relationship between serum PRDX4 and the change in pulmonary function. Fourth, cross-reaction of the anti-human PRDX4 antibody with mouse PRDX4 can be observed as the amino-acid sequences of human and mouse PRDX4 are highly homologous [25]; therefore, immunohistochemical staining of lungs of WT mice revealed human PRDX4-positive cells. Eventually, only male mice were used in this study, similar to our previous research [19, 20, 23, 25], and we were unable to evaluate the gender differences in the pathogenesis of IPF in Tg mice.

## Conclusions

The results of the present study suggest that PRDX4 is associated with the aggravation of IPF and serum PRDX4 may be useful in clinical practice of IPF patients. Further studies are warranted to enable a better understanding of the detailed role of PRDX4 in IPF.

## Supplementary information

**Additional file 1: Figure S1.** BALF PRDX4 protein levels in patients with S-IPF and AE-IPF. BALF PRDX4 protein levels did not differ significantly between patients with S-IPF and AE-IPF.

**Additional file 2: Figure S2.** The relationship between the serum and BALF PRDX4 protein levels in patients with IPF. There were no significant correlations between the serum and BALF PRDX4 protein levels (Spearman’s rank Correlation Coefficient, *r* = 0.218, *p* = 0.296).

## Data Availability

The datasets used during the current study are available from the corresponding author on reasonable request.

## Abbreviations

8-OHdG: 8-hydroxy-2’-deoxyguanosine
A-aDO_2_: Alveolar–arterial difference of oxygen
AE-IPF: Acute exacerbation of idiopathic pulmonary fibrosis
BALF: Bronchoalveolar lavage fluid
BLM: Bleomycin
CCL-2: CC chemokine ligand 2
CTGF: Connective tissue growth factor
DL_CO_: Diffusing capacity of the lungs for carbon monoxide
FVC: Forced vital capacity
GAPDH: Glyceraldehyde 3-phosphate dehydrogenase
HE: Hematoxylin and eosin
IFN-γ: Interferon γ
IL: Interleukin
IPF: Idiopathic pulmonary fibrosis
KL-6: Krebs von den Lungen-6
PaO_2_/FiO_2_: Arterial partial pressure of oxygen/fraction of inspired oxygen
PCR: Polymerase chain reaction
PDGF-B: Platelet-derived growth factor subunit B
PDGFRs: Platelet-derived growth factor receptors
PF: Pulmonary fibrosis
PRDX: Peroxiredoxin
PRDX4: Peroxiredoxin 4
S-IPF: Stable idiopathic pulmonary fibrosis
SP-D: Surfactant protein D
TGF-β1: Active tissue growth factor-β1
TNF-α: Tumor necrosis factor-α
TTF-1: Thyroid transcription factor
UIP: Usual interstitial pneumonia
α-SMA: α-smooth muscle actin
