# Supplementary figures and images for: The overexpression of peroxiredoxin-4 affects the progression of idiopathic pulmonary fibrosis
Source: BMC Pulm Med. 2019 Dec 30;19:265. doi: 10.1186/s12890-019-1032-2 (PMC6936055; doi:10.1186/s12890-019-1032-2)

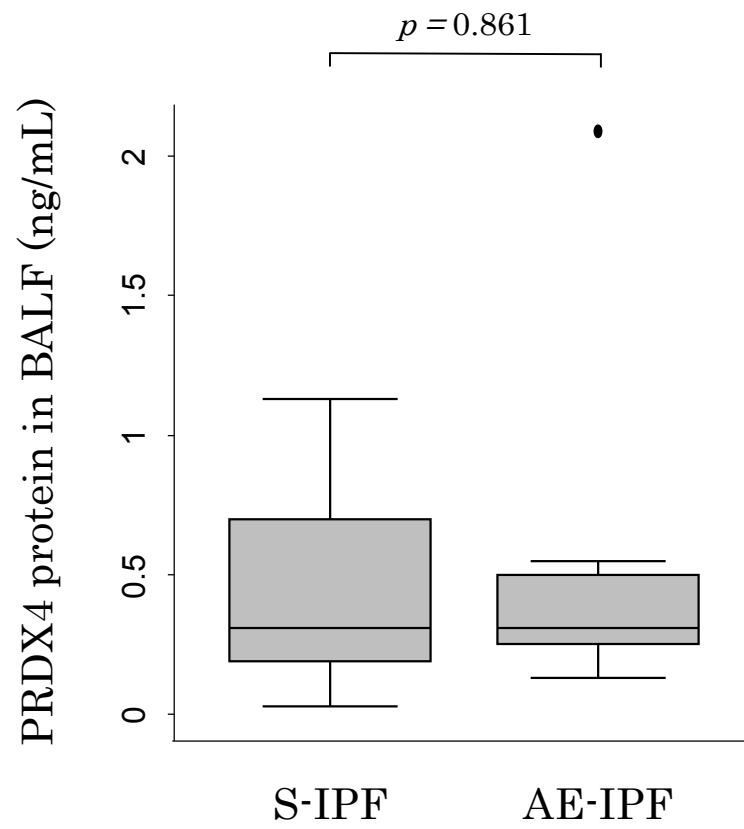

Supplement: Supplementary file 1 — Additional file 1: Figure S1. BALF PRDX4 protein levels in patients with S-IPF and AE-IPF. BALF PRDX4 protein levels did not differ significantly between patients with S-IPF and AE-IPF. [file 12890_2019_1032_MOESM1_ESM.pdf]

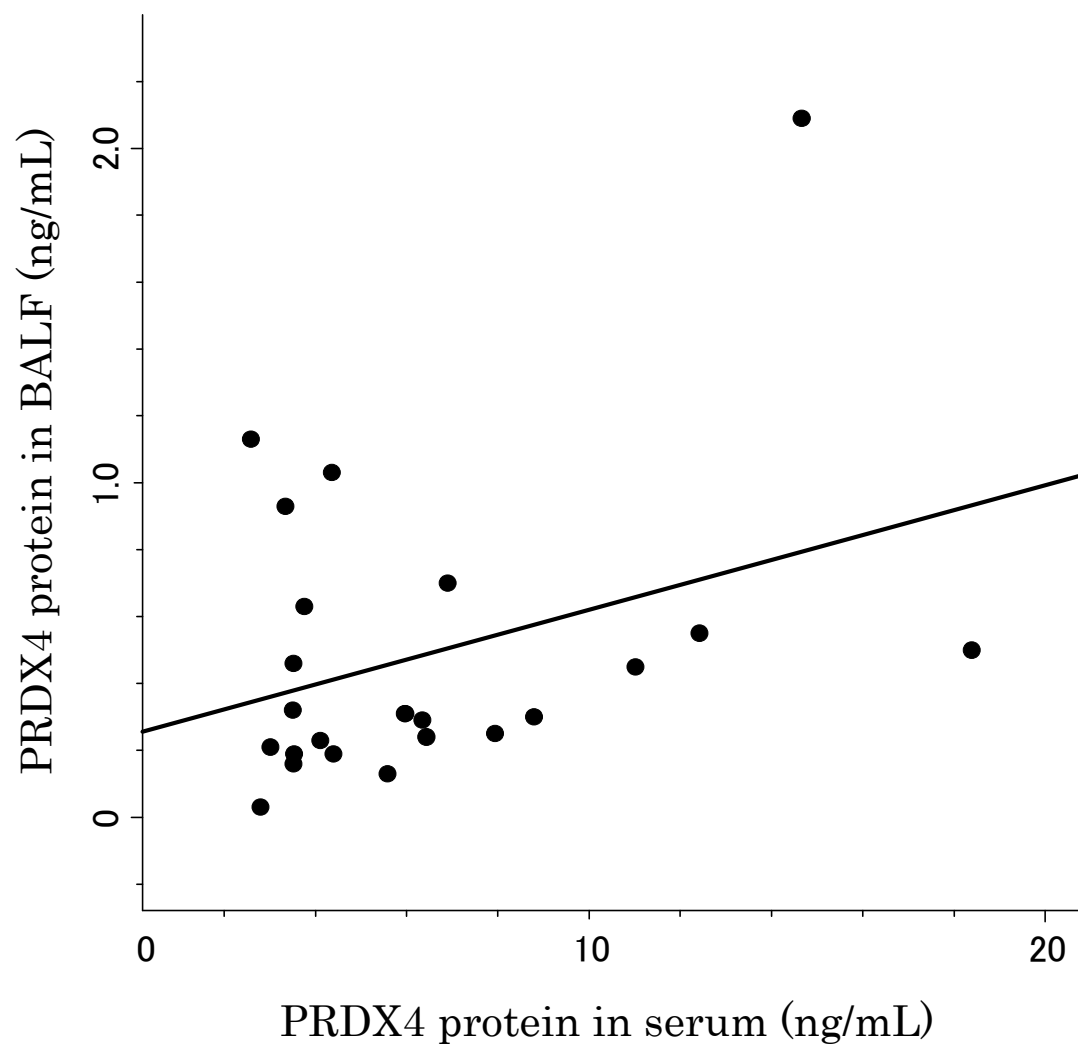

Supplement: Supplementary file 2 — Additional file 2: Figure S2. The relationship between the serum and BALF PRDX4 protein levels in patients with IPF. There were no significant correlations between the serum and BALF PRDX4 protein levels (Spearman’s rank Correlation Coefficient, r = 0.218, p = 0.296). [file 12890_2019_1032_MOESM2_ESM.pdf]
